# Transoral incisionless fundoplication and open hiatal hernia repair: A case report

**DOI:** 10.3389/fgstr.2023.1207659

**Published:** 2023-05-31

**Authors:** Anjani H. Turaga, Yasser H. Salem

**Affiliations:** ^1^ Department of Surgery, Gandhi Medical College, Hyderabad, India; ^2^ Department of Surgery, South Coast Global Medical Center, Orange County, CA, United States

**Keywords:** TIF, Hiatal Hernia, GERD, Transoral incisonless fundoplication, gastrectomy

## Abstract

**Introduction:**

Transoral incisionless fundoplication is a new procedure that has recently emerged as a potential alternative to traditional anti-reflux surgeries. It is a less invasive option with fewer complications and reduced recovery time. Hiatal hernia repair is also commonly performed in conjunction with transoral incisionless fundoplication to improve outcomes. In this case, it details a successful transoral incisionless fundoplication and hiatal hernia repair procedure in a patient with long standing gastroesophageal reflux disease (GERD). The case is unique as it involved a patient with an uncharacteristically large hiatal hernia measuring above 5cm, which is a size that is generally not considered suitable for transoral incisionless fundoplication. The hiatal hernia was repaired with a gastrectomy instead of laparoscopically due to the size and adhesions present.

**Case details:**

This case report presents an 86 year old female patient with a history of long-standing GERD symptoms from the past 10 years who had failed to respond to medical therapy. Endoscopic imaging revealed a hernia of more than 5cm in size, which was confirmed by a bravo study. A DeMeester score of 446 was reported. The patient was referred for surgery and underwent transoral incisionless fundoplication with hiatal hernia repair. Despite the large size of the hiatal hernia, it was decided to proceed with a transoral incisionless fundoplication (TIF) procedure combined with hiatal hernia repair. The gastrectomy was chosen due to significant adhesions and anatomical distortion, making it difficult to continue laparoscopically. The esophagus was fibrosed to the pericardium, and the stomach was stuck in a retrocardiac position. Laparoscopic removal of the adhesions proved difficult since the patient had friable tissues, and there was a high risk of injury to surrounding organs. The surgery was therefore converted to an open approach, and the hernia was repaired with a gastrectomy. The TIF procedure was performed successfully, and the patient had no complications postoperatively.

**Conclusion:**

This case details a successful transoral incisionless fundoplication procedure for GERD in a patient with an uncharacteristically large hiatal hernia. Despite the challenges posed by the hernia’s size and anatomical distortion, the TIF procedure combined with hiatal hernia repair was successful in providing relief from GERD symptoms, with no postoperative complications. The case highlights the potential suitability of TIF as an alternative to laparoscopic fundoplication in patients with large hiatal hernias, although gastrectomy may be necessary in cases with significant adhesions or anatomical distortion.

## Introduction

Transoral Incisionless Fundoplication (TIF) has emerged as an effective treatment for gastroesophageal reflux disease (GERD). It is a procedure which uses an endoscope and a specialized device to create a valve between the stomach and esophagus, thus preventing the reflux of gastric contents into the esophagus. This minimally invasive endoscopic technique for the management of GERD has been shown to be safe and effective in selected patients with hiatal hernias and without significant anatomical abnormalities. Case reports have demonstrated the clinical usefulness of TIF in treating symptomatic GERD patients as well. For example, a case report by Alexandru Eugen Nicolau et al. (1) documented a patient with severe GERD symptoms such as regurgitation, dysphagia, and chest pain, who underwent TIF treatment. The patient’s GERD symptoms improved significantly after the procedure, and the follow-up endoscopy showed no complications related to TIF. The patient was able to discontinue proton pump inhibitor (PPI) therapy and reported a high degree of satisfaction with the outcome.

The procedure uses a device called the “TIF device”, which is introduced transorally and uses suction to pull the esophagogastric junction (EGJ) inwards while simultaneously placing polymer fasteners to create a serosa-to-serosa plication. This creates a new valve between the stomach and esophagus, thus improving GERD symptoms. Several studies have reported on the effectiveness of TIF as a novel procedure for GERD management. One study by Phuong Huynh et al. (2) evaluated the long-term outcome of TIF in patients with GERD who had failed to respond to medical therapy. The study showed that TIF improved GERD symptoms significantly and had a low rate of side effects, demonstrating its potential as a safe and effective alternative to more invasive surgical techniques for GERD management.

Prior to this procedure, patients should undergo a comprehensive evaluation by a gastroenterologist to determine their eligibility for TIF. This includes a comprehensive laboratory workup, upper endoscopy, and imaging studies to evaluate the anatomy of the esophagus and stomach. One of the procedures that is recommended is an endoscopy procedure involving a camera, known as the Bravo camera. The EGD Bravo test involves placing a small wireless device in the esophagus to monitor acid levels over a 96-hour period, providing valuable information for proper diagnosis and management of GERD.

Hiatal hernia is a type of hernia which occurs when part of the stomach protrudes upwards into the chest through the diaphragm. Hiatal hernia repair is also often performed together with TIF in patients who have a hiatal hernia. This is done to provide better access to the esophagogastric junction and improve the success rate of TIF. Hiatal hernia repair is done laparoscopically and involves reducing the hernia and reinforcing the diaphragmatic hiatus. In most patients, an open hiatal hernia repair is only indicated in those with larger hiatal hernias that cannot be managed laparoscopically. A recent paper on hiatal hernias and TIF by Peter Janu et al. (3) reported that concurrent repair of hiatal hernia and TIF provided a significant improvement in symptoms of GERD and had a low rate of complications.

## Details of the case

This case report presents an 86 year old female patient with a history of long-standing GERD symptoms from the past 3 years who had failed to respond to medical therapy. The patient underwent a comprehensive evaluation by a gastroenterologist, which included a laboratory workup, upper endoscopy, and the Bravo camera test. A CT (computed tomography) scan without contrast was performed one year prior, which noted the presence of a large diaphragmatic hernia containing entire stomach and splenic flexure of colon without bowel obstruction. The hernia had displaced the heart anteriorly. The endoscopic findings showed a hernia measuring 8cm, 4 Hill Type valve. Esophageal candida was also noted. The Bravo test showed that the patient’s DeMeester score was 446. She had shortness of breath and a cough.

No evidence of Barrett’s esophagus was seen. Based on the results of these tests, the patient was confirmed to have a hiatal hernia and was deemed eligible for TIF. Informed consent was obtained and the patient was authorized for surgery.

**Figure 1:**
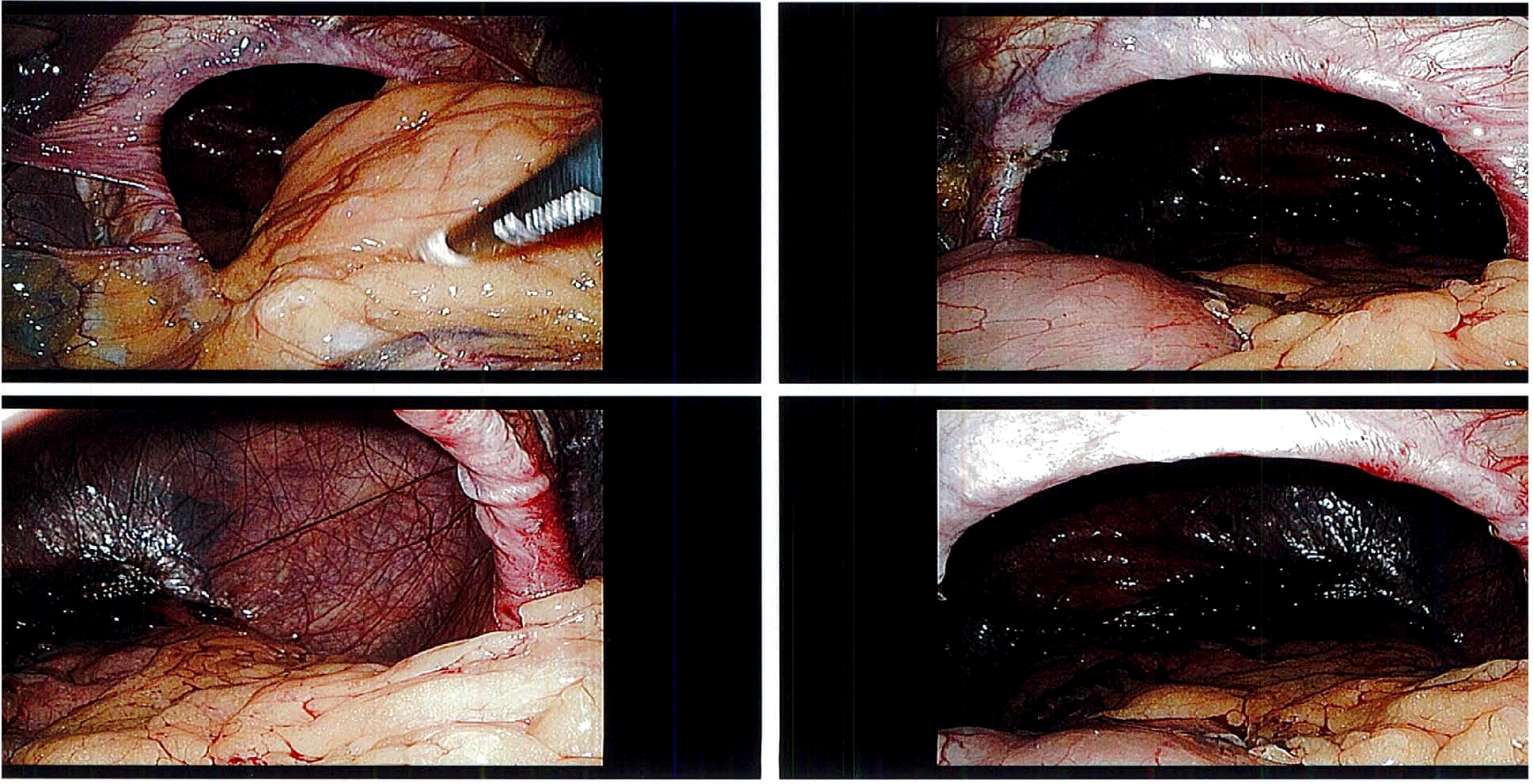
Shows initial hiatal hernia on top-left and top-right with bowel being present. Bottom-left and bottom-right show hiatal hernia after surgeon pulled herniated contents backwards laparoscopically.

The patient was scheduled for a laparoscopic hiatal hernia repair along with transoral incisionless fundoplication(TIF) on 11th April, 2023. On the day of the surgery, the patient was placed under general anesthesia and prepped and draped appropriately. On entering the patients abdomen laparoscopically, the surgeon confirmed the presence of a large hiatal hernia- measuring about 10cm in size. The patient’s esophagus, stomach, and a part of the small intestine were protruding through the diaphragmatic hiatus (**Figure 1**). The surgeon tried to reduce the hiatal sac laparoscopically, but the patient presented with significant adhesions and anatomical distortion, making it difficult to do so. The esophagus was fibrosed to the pericardium, and the stomach was stuck in a retrocardiac position. Laparoscopic removal of the adhesions proved difficult since the patient had friable tissues, and there was a high risk of injury to surrounding organs.

Given the complexity of the case and to avoid potential complications, it was decided that an open hiatal hernia repair was necessary. During the open hiatal hernia repair, the surgeon was able to reduce the herniated stomach and esophagus through a gastrotomy. Once the hiatal hernia was reduced, the surgeon reinforced the hiatus by suturing the crura together. No mesh was used to reinforce the hiatus due to potential complications.

**Figure 2:**
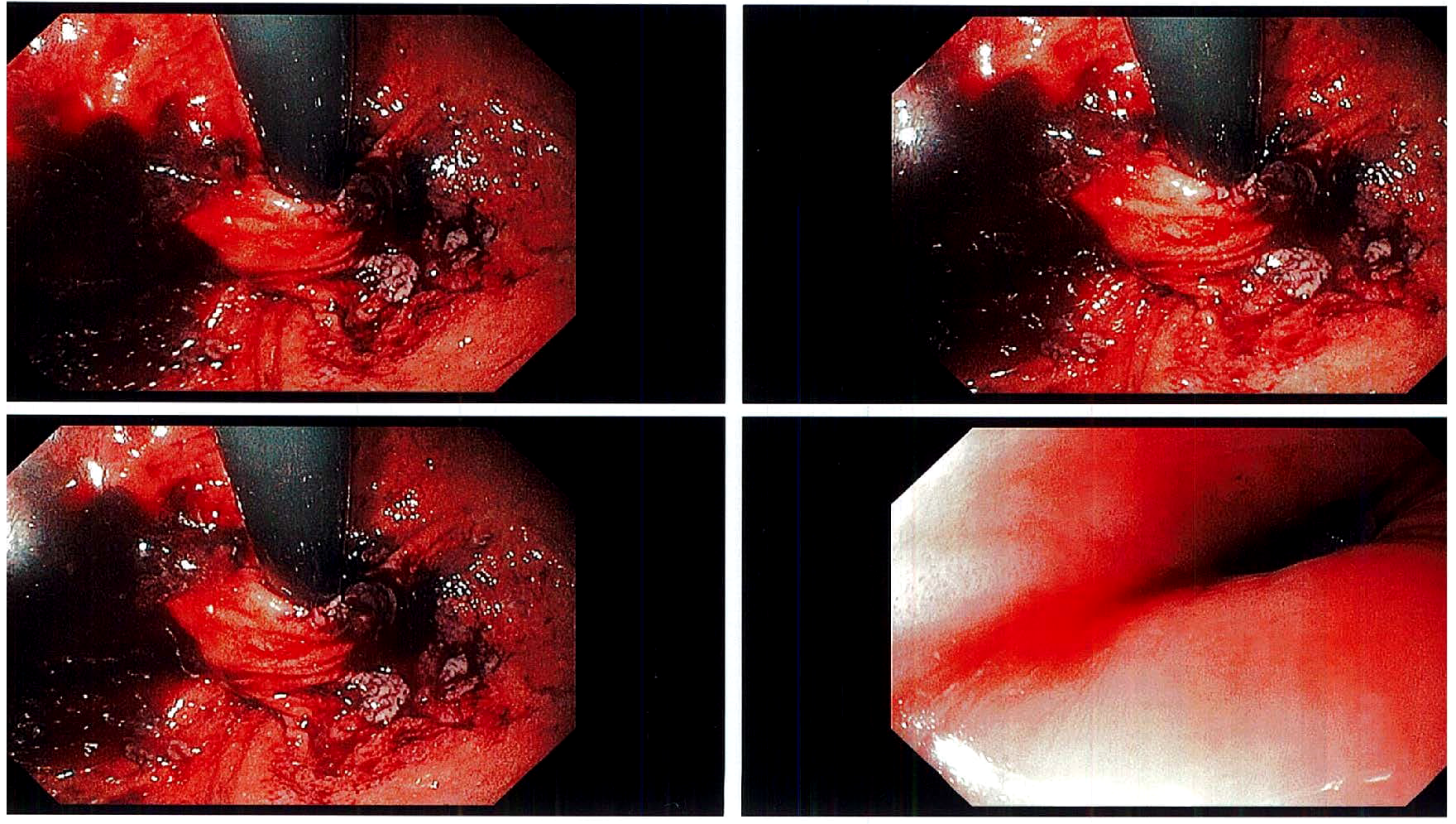
Shows tightened valve created by Esophyx device and TIF procedure on top-left, top-right, and bottom-left slides; bottom-right shows the esophagus post procedure.

Following the successful open hiatal hernia repair, the surgery proceeded with transoral incisionless fundoplication(TIF) performed *via* a transoral approach. The TIF procedure involved the use of an EsophyX device which allowed for the creation of a valve at the patient’s gastroesophageal junction without the need for any external incisions (**Figure 2**). The entire procedure was completed successfully without any perioperative complications, and the patient was discharged the next day.

The case shines a spotlight on the challenges that can arise during surgical interventions for gastroesophageal reflux disease (GERD), particularly in cases of large hiatal hernias. In such complex cases, laparoscopic approaches might not be feasible or safe, and an open approach may be required. The complexity of the case presented challenges during the surgical intervention due to significant adhesions, anatomical distortion, and friable tissues which made laparoscopic removal of the adhesions difficult and unsafe. Therefore, an open hiatal hernia repair was performed with TIF to create a valve at the gastroesophageal junction without external incisions. This dual approach was successful in reducing the hiatal hernia, reinforcing the hiatus, and creating a functional valve without any perioperative complications.

The success of the open hiatal hernia repair and transoral incisionless fundoplication (TIF) procedure in this case highlights the importance of individualized treatment plans that take into consideration the complexity of each patient’s case and their unique anatomical variations.

## Discussion

The TIF procedure replaced older methods for treating GERD such as Nissen fundoplication, which involves open or laparoscopic approaches and can be associated with higher rates of perioperative complications. Moreover, long-term use of proton pump inhibitors (PPIs), the mainstay of medical therapy for GERD, has been associated with several adverse effects, including increased risk of osteopenia, acute kidney injury, microscopic colitis and Clostridium difficile infections, as quoted by Carole E. Aubert et al. (4). Similar findings were seen in our case report, where TIF was a viable option for the patient who had comorbidities and was unwilling to take proton pump inhibitors due to concerns regarding their long-term safety.

A recent paper by Veeravich K. Jaruvongvanich et al. (5) highlights the potential benefits of TIF compared to open or laparoscopic fundoplication, including shorter hospital stays, faster recovery times, and fewer complications. In our case report, the success of the open hiatal hernia repair and TIF procedure further supports these claims. The patient was allowed to be discharged the next day, and had no perioperative complications.

Furthermore, the hiatal hernia repair using an open approach in conjunction with TIF provides a viable option for patients with large hiatal hernias in whom laparoscopic approaches may not be feasible. According to Geoffrey P Kohn et al. (6), open hiatal hernia repair can be occasionally necessary for reasons such as bleeding, splenic injury or dense adhesions, and it is important that surgeons taking these on as laparoscopic procedures are comfortable with an open repair should conversion become necessary. The patient under consideration in our case report had a large hiatal hernia and multiple comorbidities, making an open approach to the hiatal hernia repair necessary, similar to the findings of Geoffrey P Kohn et al. (6)

A large research gap exists in identifying the optimal surgical technique for treating GERD, as there are not many randomized controlled trial data to support a preference for any surgical technique. Some researchers believe that the TIF procedure is not advised for all cases, as “it cannot be performed after esophagectomy, surgical sleeve or in patients with a large hiatal hernia”, as quoted by Hiran C. Fernando (7). Contrary to this finding, in our case report, the procedure was successfully performed on a patient with a large hiatal hernia measuring 10cm.

In addition to these findings, TIF has also been shown to be efficacious and safe in the management of GERD, including in patients who have undergone peroral endoscopic myotomy (POEM) or prior fundoplication procedures, as reported in a study by Petros C Benias et al. (8). While our case report did not highlight a patient with achalasia, it does add valuable information to the literature regarding the successful use of TIF in combination with open hiatal hernia repair for GERD patients with significant adhesions around the esophagus. Therefore, TIF can have a valuable role in the management of GERD, particularly for patients who may not be suitable candidates for laparoscopic surgery or who prefer a minimally invasive approach.

In the case of this patient, the pre-operative and post-operative findings have been tabulated below to provide an understanding of how important clinical suspicion is for otherwise unexplained chronic respiratory and gastrointestinal symptoms.

## Conclusion

GERD can have detrimental effects on patients’ quality of life and overall health, and medical therapy using proton pump inhibitors may not always be effective or desirable. Our case report demonstrates the efficacy and safety of TIF in conjunction with open hiatal hernia repair for treating GERD in a patient with multiple co-morbidities and a large hiatal hernia. While further research is necessary to establish the optimal surgical technique for GERD management, TIF offers a minimally invasive alternative that can benefit patients who might not be suitable for laparoscopic surgery or want to avoid medication. In conclusion, TIF has shown to be a viable alternative to medical therapy and laparoscopic surgery in managing GERD.

## Data availability statement

The original contributions presented in the study are included in the article/Supplementary Material. Further inquiries can be directed to the corresponding author.

## Ethics statement

Written informed consent was obtained from the individual(s) for the publication of any potentially identifiable images or data included in this article.

## Author contributions

YS is an MD, FACS general surgeon who performed the surgery for the patient in this case report. AT is the medical student who wrote the case report in its entirety. All authors contributed to the article and approved the submitted version.
